# Circ_0007331 knock‐down suppresses the progression of endometriosis via miR‐200c‐3p/HiF‐1α axis

**DOI:** 10.1111/jcmm.15833

**Published:** 2020-09-22

**Authors:** Lan Dong, Lu Zhang, Hua Liu, Meiting Xie, Jing Gao, Xiaoyan Zhou, Qinghong Zhao, Silin Zhang, Jing Yang

**Affiliations:** ^1^ Department of Gynecology Renmin Hospital of Wuhan University Wuhan China; ^2^ Department of Obstetrics Renmin Hospital of Wuhan University Wuhan China; ^3^ Ultrasound Department of Obstetrics and Gynecology Renmin Hospital of Wuhan University Wuhan China; ^4^ Reproductive Medical Center Renmin Hospital of Wuhan University Wuhan China

**Keywords:** circ_0007331, endometriosis, HIF‐1α, invasion, miR‐200c‐3p, proliferation

## Abstract

Endometriosis is considered a benign gynaecological disease with cancer‐like characterizations, which has a high incidence among women of reproductive age. However, this disease has so far lacked timely diagnosis and effective treatment owing to its unclear aetiology. In this study, we identified aberrant high expression of circ_0007331 in ectopic endometrial cells by comparing the endometrial samples from patients with and without endometriosis. Further functional experiments revealed that circ_0007331 knock‐down effectively suppressed the viability, proliferation and invasive capacity of ectopic endometrial cells. Additionally, we attempted to define the molecular mechanism of circ_0007331 in the initiation and progression of endometriosis. Circ_0007331 acted as a miRNA sponge for miR‐200c‐3p to indirectly regulate the function of HIF‐1α, which plays a key role in the local angiogenesis and hypoxic mechanisms of ectopic endometrium. A final in vivo experiment confirmed that circ_0007331 knock‐down could suppress the development of endometriosis through down‐regulating the expression of HIF‐1α. Collectively, we preliminarily characterized the role and possible insights of circ_0007331/miR‐200c‐3p/HIF‐1α axis in the proliferation and invasion of ectopic endometrial cells. We hope that by exploring the potential function and molecular mechanism of circ_0007331, we can increase our biological insight into the pathogenesis of endometriosis, which will bring the new ways for the diagnosis and therapy of this disease.

## 1 INTRODUCTION

Endometriosis is considered a chronic gynaecological disorder with a high prevalence among women of reproductive age, which are usually clinically manifested by acyclic pelvic pain, dyspareunia, dysmenorrhoea and subfertility.^1^ Not only that, but endometriosis also increases the risk of other complex diseases such as migraines, painful bladder syndrome and irritable bowel syndrome.^2^ As the third leading cause of gynaecologic hospitalization in the United States, the incidence of endometriosis varies from 10% to 40% with the increase of related concurrent symptoms.^3^ Although several theories including Sampson's theory of retrograde menstruation have been proposed, the pathogenesis of this disease is not thoroughly understood.^4^ Besides, the clinical diagnosis of endometriosis is also extremely rough and hysteretic. Due to the lack of adequately sensitive and specific signs as well as blood tests, the current gold standard for the diagnosis of endometriosis mainly consists of invasive laparoscopic surgery and subsequent histopathological examination, which will cause a delay in disease confirmation and bring great pain to patients.^5^ Compared with traditional surgical diagnosis, non‐invasive or minimally invasive biomarkers not only help to shorten the diagnosis delay and improve patient compliance, but also have the advantages of low risk and low cost. Therefore, the World Endometriosis Society (WES) and the World Endometriosis Research Foundation (WERF) have identified the development of new non‐invasive biomarkers as scientific issues that urgently need to be solved in this disease field.^6^

Circular RNAs (circRNAs) are a type of regulatory RNAs that lack canonical 5′ cap and 3′ poly‐A tail attributed to their covalent closed‐loop structures.^7^ As the competitive endogenous RNAs, circRNAs regulate the expression of multiple genes at the transcriptional and post‐transcriptional levels by sponging downstream microRNA (miRNA). Most meaningfully, circRNAs have been increasingly used as the biomarker in the diagnosis, therapy and prognosis of various diseases due to its advantages of abundant expression level, high stability and covalent terminals.^8^ Circ _0081001 is identified as a potential biomarker for the diagnosis of osteosarcoma, which can dynamically monitor and reflect the disease development of the patients.^9^ Hsa_circ_0000291 is regarded as a promising therapeutic target for gastric cancer (GC) due to its effective inhibitory role on the metastasis and growth of GC cells by being silenced or down‐regulated.^10^ Circ_0079593 predicts an adverse prognosis owing to its strong association with the cell growth and invasion of glioma.^11^ The research of circRNAs in endometriosis has also made preliminary progress. Analysis of clinical samples by microarray and bioinformatics methods revealed 88 differentially expressed circRNAs between eutopic and normal endometrium, of which 11 were up‐regulated and 77 were down‐regulated.^12^ In addition, circ_103470 and circ_101102 were found to regulate epithelial‐mesenchymal transition (EMT) of endometriosis through sponge miR‐141‐5p.^13^ Further functional analysis showed that downstream mRNAs of these differentially expressed circRNAs are mainly involved in immune‐inflammatory response and cell cycle regulation. Obviously, the potential of circRNAs as a biomarker to monitor the initiation and progression of diseases is also applicable to endometriosis.

Although a series of differentially expressed circRNAs have been widely discovered in various diseases, little is known about their biogenesis processes and underlying mechanisms. It has been learned that the regulatory function of circRNAs mainly depends on its directly bound downstream miRNAs.^14^ MicroRNAs (miRNAs) are a type of conservative small regulatory non‐coding RNAs that can suppress the expression of target genes. As miRNAs are involved in key biological processes relied on cell life such as cell proliferation, apoptosis, development and differentiation, they are considered as vital regulators of gene expression. Circ_0000285 is reported to interact with miR‐654‐3p to act as a miRNA sponge which in turn activates MAPK6 in diabetic nephropathy, thereby causing podocyte damage.^15^ MiR‐25‐3p could affect the unchecked proliferation of endometrium in endometriosis by directly regulating specificity protein 1 (Sp1).^16^ Recently, a report delving into the molecular basis of the circRNA/miRNA network in the pathogenesis of endometriosis found that hsa_circ_0067301/miR‐141‐5p/Notch‐1 axis promotes the EMT process of this disease.^17^

In this study, we identified aberrant high expression of circ_0007331 in endometriosis by comparing endometrial tissue samples from patients with endometriosis and normal individuals. Further in vivo and in vitro experiments demonstrated the presence of the circ_0007331/miR‐200c‐3p/HIF‐1α axis in the positive regulation of ectopic endometrial cell viability, proliferation and invasion. We hope that by exploring the potential function and molecular mechanism of circ_0007331, we can increase our biological insight into the pathogenesis of endometriosis, which will bring the new ways for the diagnosis and therapy of this disease.

## 2 MATERIALS AND METHODS

Ethical approvals were obtained from the Ethics Committee of the Renmin Hospital of Wuhan University. All collected samples were obtained subsequent to receive full written informed consent from the patients. Relevant animal experiments were conducted complied with the institutional ethical guidelines.

### 2.1 Clinical samples collection

All samples were taken from female patients aged 35‐45 with a regular menstrual cycle (25‐32 days). The collection time was the proliferative phase of the menstrual cycle, and patients did not receive any hormonotherapy for at least 6 months pre‐operatively. Endometriosis (EM) samples were collected from 25 patients with ovarian chocolate cysts treated by hysteroscopy and laparoscopy surgery. These endometriosis patients were identified as stage 3 or 4 as stated in the classification of the American Society of Reproductive Medicine. Control samples were collected from an equal number of women without endometriosis. Above tissue samples were immersed in liquid nitrogen immediately after surgical excision for quick freezing and stored at −80°C until mRNA extraction experiments were conducted.

### 2.2 Cell culture and identification

The experimental cell lines in this study were divided into normal endometrial (NE) cells and eutopic endometrial (EE) cells, which derived from the 0.5‐1 mm thick part of the endometrial functional layer of patients without endometriosis and patients with ovarian chocolate cysts, respectively. Immediately after the samples were gathered, they were cultured in sterile saline containing 1.5% antibiotics and antifungal. Afterwards, these tissues were washed, minced, digested with collagenase and trypsin and filtered with a 50‐µm‐diameter nylon mesh to obtain the desired cells. Cells were grown in Dulbecco's modified Eagle's medium/Nutrient Mixture F‐12 Ham (DMEM/F12, Invitrogen) replenished with 10% foetal bovine serum (FBS, Sigma‐Aldrich) in a humidified incubator containing 5% CO2 at 37°C.

### 2.3 Cell transfection

The knock‐down treatments are required for evaluating the functions of circ_0007331 in EE cells. Therefore, the corresponding transfection of EE cells is necessary to knock‐down circRNA or miRNA. To generate circ_0007331 or miR‐200c‐3p knock‐down model, shRNA‐negative control (sh‐NC), shRNA circ_0007331 (sh‐Circ) and anti‐miR‐200c‐3p were all synthesized and obtained by Sangon Biotech. The circ_0007331 shRNA, shRNA NC and anti‐miR‐200c‐3p were constructed as follows: 5′‐AAATTTTCACACAGGCAGCTT‐3′, 5′‐UUCUCCGAACGUGUCACGUUU‐3′ and 5′‐UCCAUCAUUACCCGGCAGUAUUA‐3′, respectively. Then, the sequences were, respectively, transfected into EE cells with the 25 nM oligonucleotides by utilizing Lipofectamine 3000 reagent (Invitrogen) in accordance with the manual of producer. These cells were harvested for subsequent investigations after transfection for 60 hours. Afterwards, EE cells were screened with 2 µg/mL puromycin (Sigma‐Aldrich) for two weeks as reported by the manufacturer's instructions.

### 2.4 Quantitative reverse transcription polymerase chain reaction (qRT‐PCR)

The mRNA expressions of circ_0007331, miR‐200c‐3p and HIF‐1α in the experimental groups were examined by qRT‐PCR. In strict accordance with the manufacturer's guidelines, total RNAs were extracted from EE cells by using TRIzol reagent (Thermo Fisher Scientific). For circRNA, cDNA was reverse‐transcribed by the QuantiTect® Reverse Transcription kit (Qiagen) and quantification was evaluated with an SYBR Green PCR Kit (SYBR® Green Realtime PCR Master Mix; Toyobo). For miRNA, QuantiTect® Reverse Transcription kit was employed to reverse the total RNAs and SYBR Green PCR Kit was applied to examine the mRNA expression of miR‐200c–3p. The qRT‐PCR primers were obtained from Sangon Biotech. The sequences of the primers used were listed below: Forward, 5′‐GAATGGGATTCGAGACCTG‐3′ and reverse, 5′‐TTCTTCCAAAGCTGCCTGT‐3′ for circ_0007331; forward, 5′‐GGGGTAGGGGAAGGTGGTTTA‐3′ and reverse, 5′‐CACCACCCCAATCCCTAAAAACACT‐3′ for miR‐200c‐3p; forward, 5′‐CAGCAACGACACAGAAACTG‐3′ and reverse, 5′‐AAAGTTCCAGTGACTCTGGA‐3′ for HIF‐1α.

### 2.5 Western blot analysis

All HIF‐1α proteins were extracted from the endometrial cells lysed in RIPA lysis buffer (RIPA, Beyotime), and the protein concentrations were evaluated by bicinchoninic acid (BCA) analysis (Beyotime). The same amounts of protein were separated using 10% SDS‐PAGE and transferred onto polyvinylidene fluoride membranes (Millipore). Then, the membranes were blocked using 5% skim milk powder at room temperature for 1 hour, followed by incubation with primary antibody against HIF‐1α (Catalogue#3716, Cell Signaling technology) at 4°C overnight. The next day, membranes were then conjunct with secondary antibodies (1:5000) at room temperature for 1 hour. Finally, every protein band was visualized by an enhanced chemiluminescence kit (Pierce Biotechnology; Thermo Fisher Scientific, Inc) and related data were quantified by Image Lab Software.

### 2.6 Cell counting kit‐8 (CCK‐8) assay

The viability of EE transfected cells was detected by the CCK‐8 method. Cells were inoculated into 96‐wells with a density of 5000 cells per well and incubated until cell attachment. Then, these seeded cells were cultured for 1, 2, 3, 4 and 5 days separately after transfection. Following treatment, 10 µL of the CCK‐8 solution (KeyGen) was supplied to every well. Finally, a microplate spectrophotometer (BioTek) was employed to detect the OD value of each well at 450 nM.

### 2.7 EdU assay

The effects on the proliferation of EE cells were determined by EdU assay using the BeyoClick™ EdU‐555 Kit (Beyotime Biotechnology) in line with the protocols of manufacturer. Transfected EE cells were seeded in 96 wells and cultured for 2 days. After fixed with 4% paraformaldehyde, cells were added with 5‐ethynyl‐2'‐deoxyuridine (EdU, 10 µM each) and cultivated at 37°C for 2 hours. These cells were subsequently stained using Apollo Dye Solution and DAPI. Finally, the EdU‐positive cells were visualized and analysed through the fluorescence microscope (Olympus).

### 2.8 Transwell invasion assay

Cell invasion ability was analysed by transwell assay using BioCoat Matrigel invasion chambers (BD Biosciences). Transwell chambers were settled in a sterile 24‐well cell culture plate initially. The EE transfected cells were added to FBS‐free DMEM to prepare cell resuspension with a density of 2.5 × 105 cells/mL. 100 µL of the above cell suspension added onto the top chamber and 600 µL of culture medium containing 10% FBS were seeded to the under chamber, respectively. Then, these treated cell culture plates were incubated with 5% CO2 at 37°C for 36 hours. Afterwards, the cells that migrated to the lower chamber were fixed with 70% MeOH and stained with 0.5% crystal violet in turn. Finally, the number of migrated cells was determined by microscopic counting.

### 2.9 Luciferase reporter assay

The pGL3‐basic vectors (GenePharma) with either circ_0007331‐WT or circ_0007331‐MUT were co‐transfected with miR‐200c‐3p mimic or mimic NC into HEK 293T cells utilizing Lipofectamine 2000 reagent (Thermo Fisher). Correspondingly, the pGL3‐basic vectors with either HIF‐1α‐WT or HIF‐1α‐MUT were co‐transfected with miR‐200c‐3p mimic or mimic NC into HEK 293T cells utilizing Lipofectamine 2000 reagent. The luciferase activities were measured by the dual‐luciferase assay system after 48 hours.

### 2.10 In vivo treatment

Eighteen 8‐week‐old female C57BL6 mice were housed in our animal centre for 1 week after purchased from Shanghai Animal Centre. The endometriotic mouse model was established through implanting mouse endometrial fragments into the abdominal cavity of the above mice. Then, these mice were divided into three groups of 6 each, which were injected intraperitoneally with circ_0007331 shRNA, shRNA NC and anti‐miR‐200c‐3p by the in vivo‐jet PEI delivery agent (Nanoeast Biotech), respectively. Endometriotic cysts required for subsequent experiments were taken from mice sacrificed after 4 weeks. The lesion volumes were calculated using the formula (largest length) × (smallest width) 2 × ½.

### 2.11 Immunohistochemistry

After fixed in 4% paraformaldehyde, embedded in paraffin and sliced successively, the mouse model samples were incubated with the corresponding primary antibodies against HIF‐1α (Catalogue#3716, Cell Signaling technology) at 4°C for 24 hours. The intensity and percentage of staining were appraised by H‐score (a semi‐quantitative grading system). The images were captured and analysed using an optic microscope (Olympus).

### 2.12 Statistical analysis

The results of each experiment were repeated three times, and the experimental data were represented as means ± standard deviation (SD). The analyses were mainly interpreted applying with the software statistical SPSS version 19.0 (IBM). The statistical variances between the control group and experimental group were calculated through the unpaired two‐tailed t test. Other statistical variances among multigroups were assessed by utilizing one‐way ANOVA or two‐way ANOVA. A *P* value of less than .05 (*P* < .05) was determined as statistically significant.

## 3 RESULTS

### 3.1 The expression of circ_0007331 is abnormally elevated in endometriosis

**FIGURE 1:**
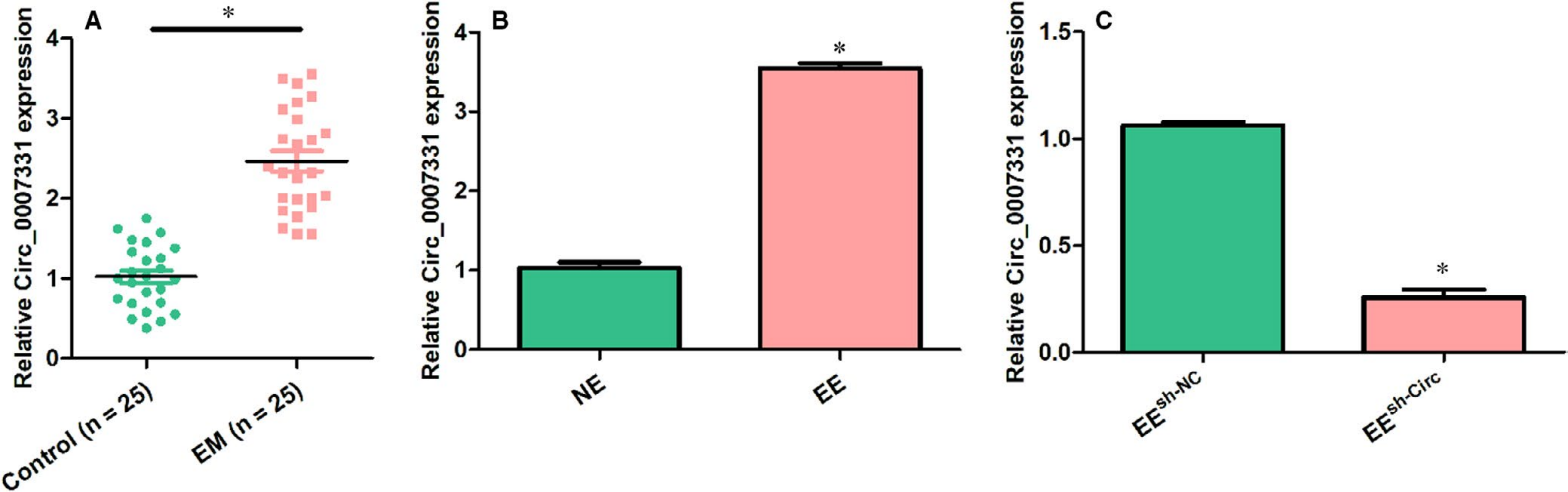
The expression of circ_0007331 is abnormally elevated in endometriosis. (A) The mRNA levels of circ_0007331 in the endometriosis (EM) patients and healthy controls were evaluated by using qRT‐PCR. (B) The mRNA levels of circ_0007331 in the ectopic endometrial (EE) cells and the normal endometrial (NE) cells were detected by using qRT‐PCR. (C) The circ_0007331 knock‐down model was constructed by transfecting circ_0007331 shRNA or shRNA NC in EE cells. N = 3 for each experiment and the data were presented as mean ± SD. *P < .05

To preliminarily explore the possible relevance between circ_0007331 and endometriosis, we detected the mRNA levels of circ_0007331 in endometria by using endometrial specimens from 25 endometriosis (EM) patients and 25 normal individuals through qRT‐PCR. The results showed that circ_0007331 expressed exceptionally high in endometriosis compared to the normal control group (Figure 1A). Besides, the expression level of circ_0007331 in ectopic endometrial (EE) cells from patients with endometriosis also significantly increased compared with the normal endometrial (NE) cells from those patients without endometriosis as well as other endometrial diseases (Figure 1B). To further evaluate the effect of circ_0007331 in the initiation and progression of endometriosis, the shRNA circ_0007331 or shRNA NC was designed and transfected into EE cells to knock‐down the expression of circ_0007331 (Figure 1C). The above results indicated that circ_0007331 abnormally elevated in endometriosis and might play a potential role in the biological processes of the endometrium.

### 3.2 Circ_0007331 knock‐down results in decreased cell proliferation and invasion of the EE cells in endometriosis

**FIGURE 2:**
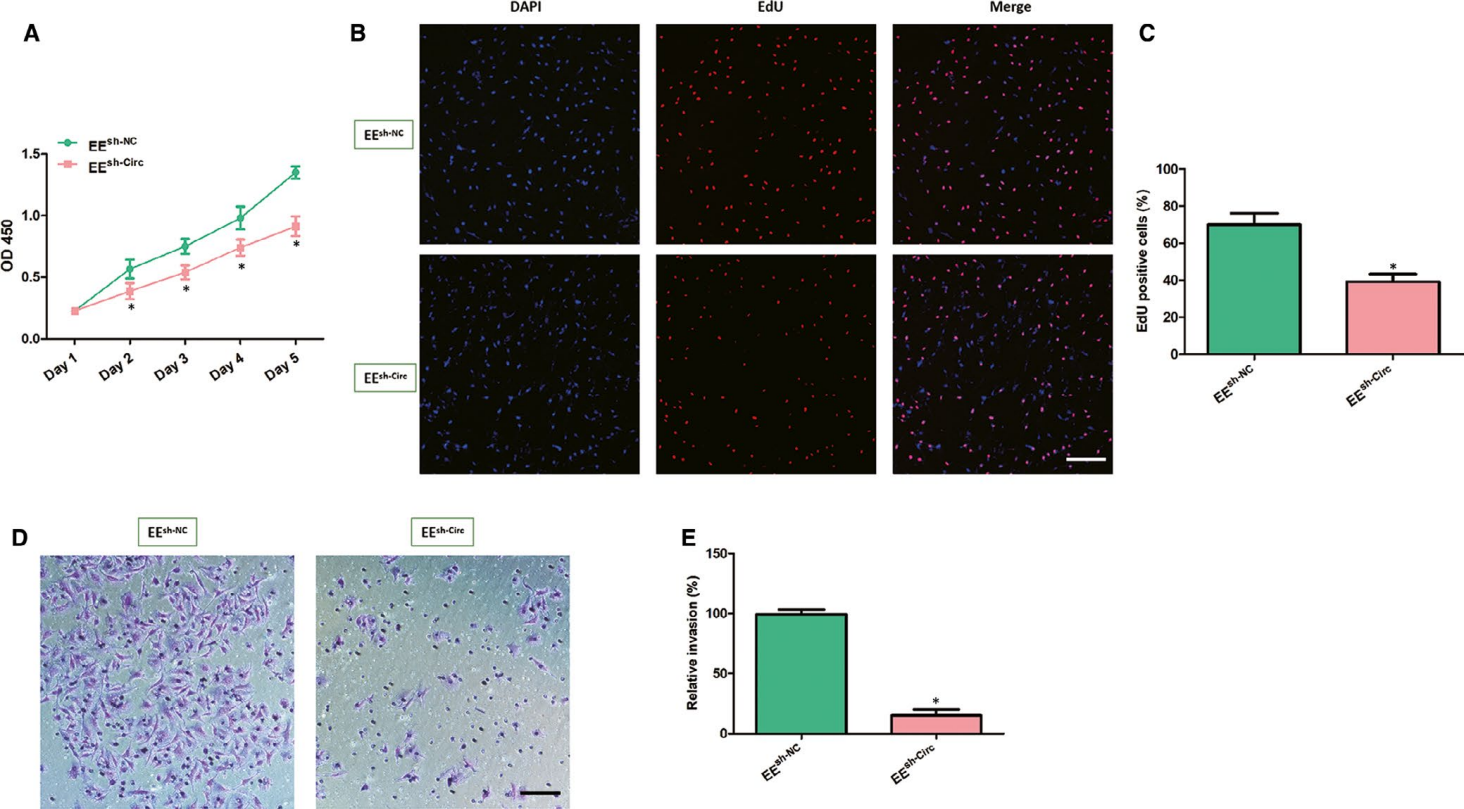
Circ_0007331 knock‐down results in reduced cell proliferation and invasion of the EE cells in endometriosis. A, The growth curves of transfected EE cells were detected by CCK‐8 assay at each indicated time‐points (1, 2, 3, 4 and 5 d). B, C, The proliferative capacity of transfected EE cells was evaluated by EdU assay (Scale bar = 100 μm). D, E, The invasiveness of transfected EE cells was evaluated by transwell invasion assay (Scale bar = 50 μm). N = 3 for each experiment and the data were presented as mean ± SD. *P < .05

We first determined the effects of circ_0007331 on the viability and proliferation of EE cells. The CCK‐8 assay was employed to appraise the cell growth of EE cells following circ_0007331 knock‐down. The cell growth curve of EE cells transfected with shRNA circ_0007331 displayed significantly lower OD450 value compared with those shRNA NC transfection cells, demonstrated that down‐regulation of circ_0007331 expression level dramatically suppressed EE cells growth (Figure 2A). The proliferation of these transfected EE cells was also detected through EdU incorporation assay. The results of fluorescence images and counting showed that circ_0007331 knock‐down led to a considerable decrease in the number of EdU‐positive cells compared with the shRNA NC group (Figure 2B,C). Besides, the function of circ_0007331 in the invasive ability of EE cells was also measured utilizing the Transwell invasion assay. Representative staining images and statistical analysis presented that the number of EE cells passing through the chambers significantly dropped off after circ_0007331 knock‐down, whereas the shRNA NC transfected cells still maintained highly invasive (Figure 2D,E). These evidence suggested that circ_0007331 predictably acted a positive part in the viability, proliferation and invasion of EE cells.

### 3.3 Circ_0007331 knock‐down suppresses the proliferation of EE cells by down‐regulating the expression of HIF‐1α

**FIGURE 3:**
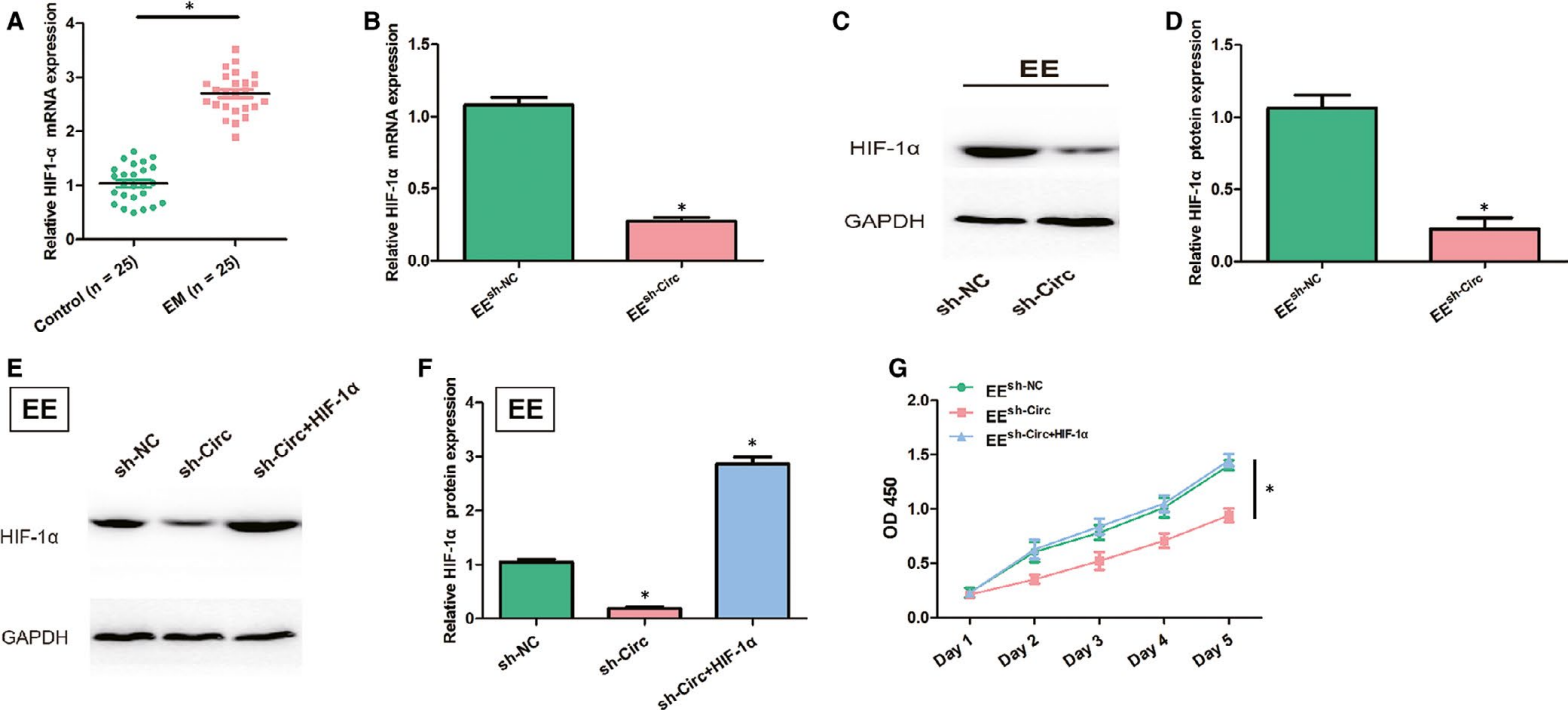
Circ_0007331 knock‐down suppresses the proliferation of EE cells by down‐regulating the expression of HIF‐1α. A, The mRNA levels of HIF‐1α in the EM patients and healthy controls were evaluated by using qRT‐PCR. B, The mRNA levels of HIF‐1α in the transfected EE cells were detected by qRT‐PCR. C, D, The protein levels of HIF‐1α in the transfected EE cells were detected by Western blot. E, F, The protein levels of HIF‐1α in the transfected EE cells were detected by Western blot after application of HIF‐1α stabilizer. G, The growth curves of transfected EE cells were detected by CCK‐8 assay after application of HIF‐1α stabilizer. N = 3 for each experiment and the data were presented as mean ± SD. *P < .05

Given that the strong connection between HIF‐1α and the adhesion as well as the proliferation of endometrial cells in endometriosis, we explored the effect of circ_0007331 on the expression of HIF‐1α in this disease. Similar to the change of circ_0007331, the mRNA level of HIF‐1α significantly rose in endometriosis (Figure 3A). Besides, circ_0007331 knock‐down caused the double reduction of HIF‐1α both at mRNA and protein levels in EE cells (Figure 3B‐D). These results indicated a possible regulation of HIF‐1α by circ_0007331 in endometriosis. We next applied the HIF‐1α stabilizer to verify this speculation. Compared with simply down‐regulating circ_0007331, supplementation of HIF‐1α stabilizers markedly increased the protein level of HIF‐1α in EE cells (Figure 3E,F). Meanwhile, the loss of EE cell viability caused by circ_0007331 knock‐down was also enhanced with the restoration of HIF‐1α expression (Figure 3G). Apparently, the suppression of EE cell proliferation by circ_0007331 knock‐down requires the cooperation of downstream‐regulated protein HIF‐1α.

### 3.4 MiR‐200c‐3p is predicted to mediate the regulation of HIF‐1α by circ_0007331

**FIGURE 4:**
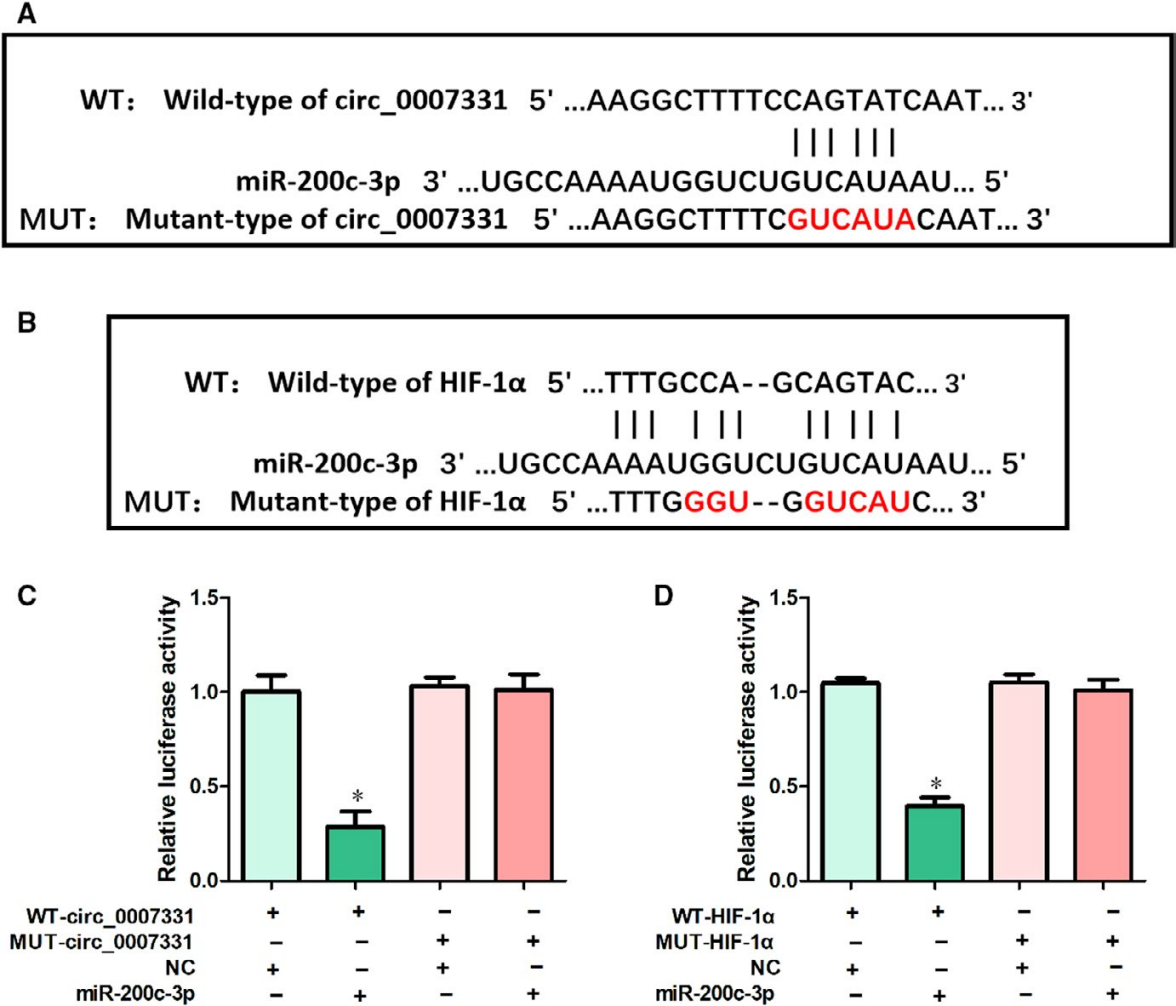
MiR‐200c‐3p is predicted to mediate the regulation of HIF‐1α by circ_0007331. (A) The inferred binding regions between circ_0007331 and miR‐200c‐3p. (B) The inferred binding regions between HIF‐1α and miR‐200c‐3p. (C) Luciferase activities were evaluated in circ_0007331 WT or Mut with miR‐200c‐3p mimic or mimic NC co‐transfected cells. (D) Luciferase activities were evaluated in HIF‐1α WT or Mut with miR‐200c‐3p mimic or mimic NC co‐transfected cells. N = 3 for each experiment and the data were presented as mean ± SD

CircRNAs are known to regulate the downstream gene through sponging the corresponding miRNAs. We therefore next committed to searching the miRNAs that link circ_0007331 and HIF‐1α to refine this regulatory network. With the help of Arraystar's home‐made miRNA target prediction software based on TargetScan (http://www.targetscan.org/vert_71) and miRanda (http://www.microrna.org/microrna), we found that the seed region of miR‐200c‐3p has both complementary sequences to circ_0007331 and HIF‐1α (Figure 4A,B). To validate the website prediction, we performed the dual‐luciferase reporter assay. The results illuminated that overexpressing miR‐200c‐3p effectively inhibited the luciferase activity of wild‐type (WT) circ_0007331 instead of mutant (MUT) (Figure 4C). Similarly, the enhanced expression of miR‐200c‐3p also selectively inhibited the luciferase activity of WT HIF‐1α (Figure 4D). Collectively, these revealed that miR‐200c‐3p possessed the physical basis for mediating the regulation of HIF‐1α by circ_0007331.

### 3.5 Circ_0007331 affects the proliferation and invasion of EE cells by sponging miR‐200c‐3p to regulate HIF‐1α

**FIGURE 5:**
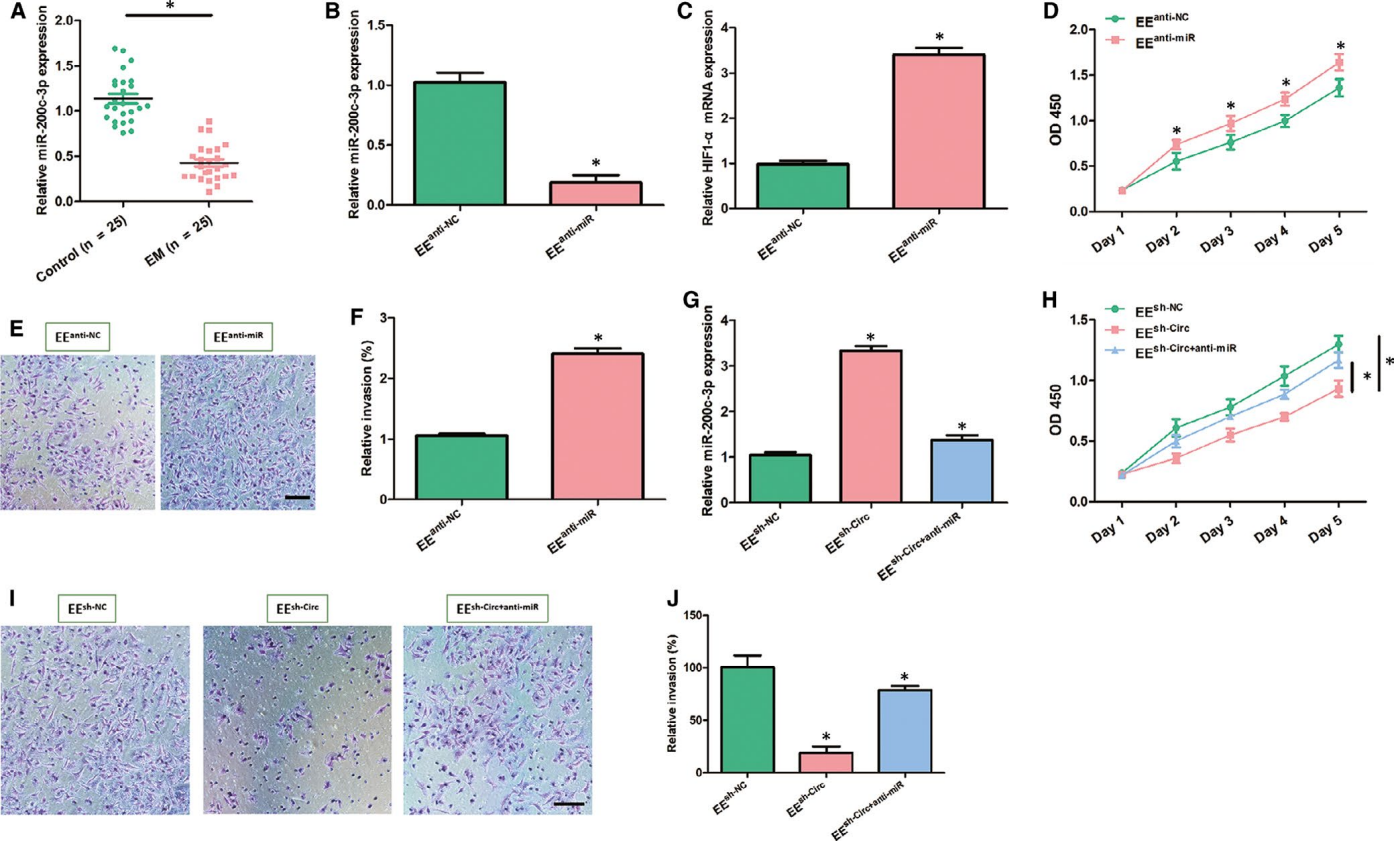
Circ_0007331 suppresses the proliferation and invasion of EE cells by sponging miR‐200c‐3p to regulate HIF‐1α. A, The mRNA levels of miR‐200c‐3p in the EM patients and healthy controls were evaluated by using qRT‐PCR. B, The expression levels of miR‐200c‐3p were detected by qRT‐PCR in the EE cells transfected with anti‐miR‐200c‐3p or anti‐NC. C, The mRNA levels of HIF‐1α were detected by qRT‐PCR in the EE cells transfected with anti‐miR‐200c‐3p or anti‐NC.D, The growth curves of EE cells were detected by CCK‐8 assay at each indicated time‐points (1, 2, 3, 4 and 5 d) after transfected with anti‐miR‐200c‐3p or anti‐NC. E, F, The invasiveness of EE cells was evaluated by transwell invasion assay after transfected with anti‐miR‐200c‐3p or anti‐NC (Scale bar = 50 μm). G, The expression levels of miR‐200c‐3p in EE cells were detected by qRT‐PCR after transfected with shRNA NC or circ_0007331 shRNA or circ_0007331 shRNA and anti‐miR‐200c‐3p. H, The growth curves of EE cells were detected by CCK‐8 assay at each indicated time‐points (1, 2, 3, 4 and 5 d) after transfected with shRNA NC or circ_0007331 shRNA or circ_0007331 shRNA and anti‐miR‐200c‐3p. I, J, The invasiveness of EE cells was evaluated by transwell invasion assay after transfected with shRNA NC or circ_0007331 shRNA or circ_0007331 shRNA and anti‐miR‐200c‐3p (Scale bar =50 μm). N = 3 for each experiment and the data were presented as mean ± SD. *P < .05

Likewise, we also evaluated the changes of miR‐200c‐3p in endometriosis. Contrary to the results of circ_0007331, the mRNA levels of miR‐200c‐3p dropped off significantly in the endometrial specimens from 25 EM patients compared with the other 25 normal individuals (Figure 5A). When the expression of miR‐200c‐3p was forced down in EE cells, the proliferation and invasion ability of the cells as well as the mRNA level of HIF‐1α all presented a markedly increase (Figure 5B‐F). Moreover, the reduction of miR‐200c‐3p expression and the weakening of EE cell proliferation and invasion ability caused by circ_0007331 knock‐down were all restored to a certain extent after suppressing miR‐200c‐3p (Figure 5G‐J). The above data provide biological evidence that the circ_0007331/miR‐200c‐3p/HIF‐1α axis regulates EE cell proliferation and invasion in endometriosis.

### 3.6 Circ_0007331 knock‐down suppresses the progression of endometriosis via miR‐200c‐3p/HIF‐1α axis in vivo

**FIGURE 6:**
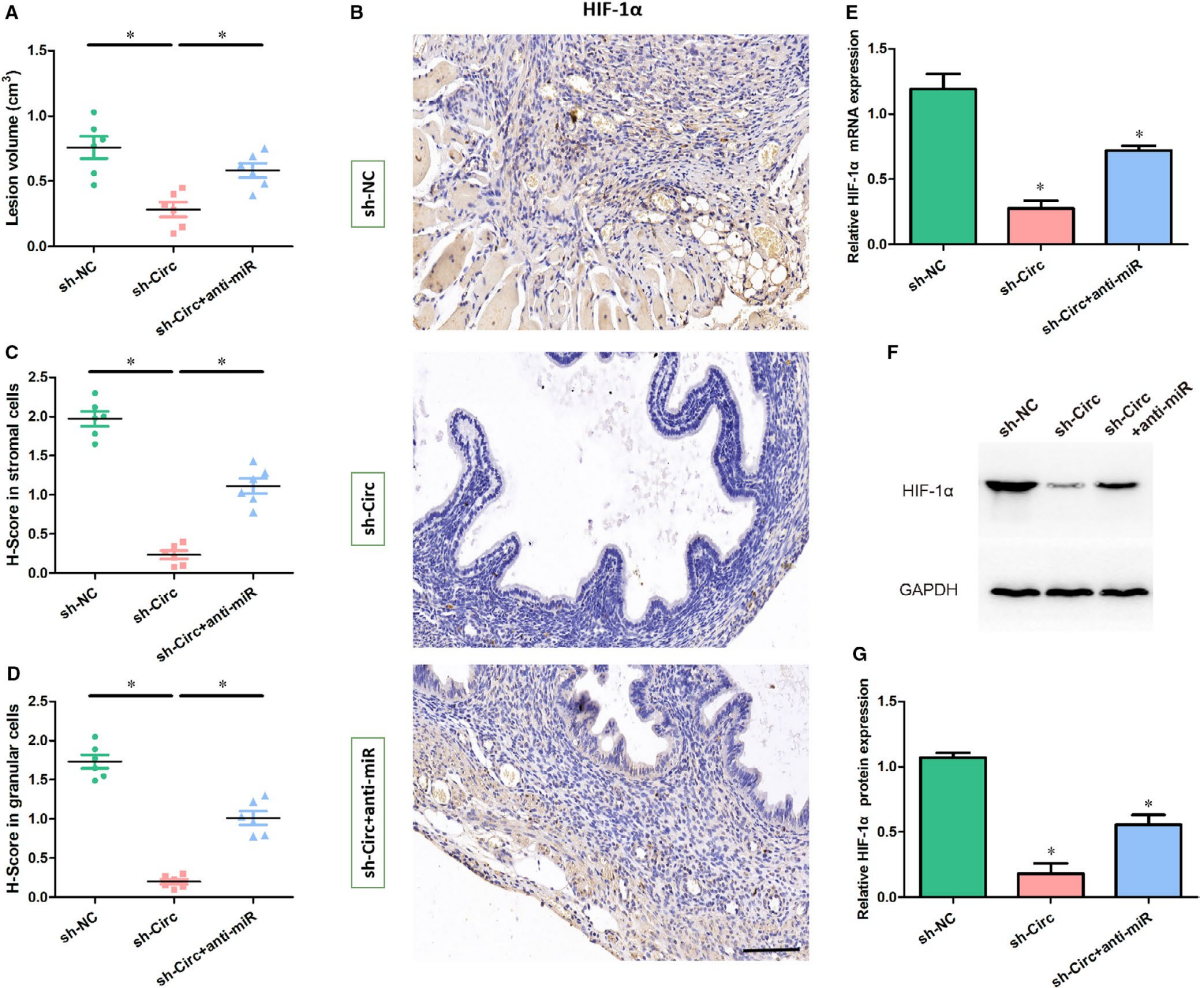
Circ_0007331 knock‐down suppresses endometriosis progression in vivo through down‐regulating the mRNA level and protein level of HIF‐1α. (A) Scatter plot shows the quantification of lesion volumes from the mice treated with shRNA NC or circ_0007331 shRNA or circ_0007331 shRNA and anti‐miR‐200c‐3p. (B) Representative immunohistochemistry photomicrographs of HIF‐1α expression in the mice lesions treated with shRNA NC or circ_0007331 shRNA or circ_0007331 shRNA and anti‐miR‐200c‐3p (Scale bar = 50 µm). (C) Scatter plot shows the expression of HIF‐1α in stromal cells from the mice lesions treated with shRNA NC or circ_0007331 shRNA or circ_0007331 shRNA and anti‐miR‐200c‐3p by H‐score (*y*‐axis). (D) Scatter plot shows the expression of HIF‐1α in glandular cells from the mice lesions treated with shRNA NC or circ_0007331 shRNA or circ_0007331 shRNA and anti‐miR‐200c‐3p by H‐score (*y*‐axis). (E) The mRNA levels of HIF‐1α were detected using qRT‐PCR in the mice lesions treated with shRNA NC or circ_0007331 shRNA or circ_0007331 shRNA and anti‐miR‐200c‐3p. (F, G) The protein levels of HIF‐1α were detected using Western blot in the mice lesions treated with shRNA NC or circ_0007331 shRNA or circ_0007331 shRNA and anti‐miR‐200c‐3p. N = 3 for each experiment and the data were presented as mean ± SD. N = 6 mice for each group. *P < .05

To further clarify the connection of circ_0007331, miR‐200c‐3p and HIF‐1α in the endometriosis progression, we established endometriosis model mice by implanting mouse endometrial fragments into the abdominal cavity, following treated with circ_0007331 shRNA, shRNA NC and anti‐miR‐200c‐3p. Four weeks later, a series of biochemical analyses on the classical endometriosis‐like lesions formed in mice were performed. Scatter plot shows circ_0007331 knock‐down could effectively reduce the lesion sizes while miR‐200c‐3p suppression counteracts this beneficial effect (Figure 6A). Immunohistochemistry results showed that stromal cells and glandular cells from circ_0007331 knock‐down mice were negative for HIF‐1α, whereas anti‐miR‐200c‐3p treatment recovered its expression level (Figure 6B‐D). Moreover, the quantitative analysis of mRNA and protein content of HIF‐1α in pathological cells was carried out via qRT‐PCR and Western blot. Similar to the above immunohistochemical consequence, both mRNA level and protein level of HIF‐1α were significantly down‐regulated after circ_0007331 knock‐down, while anti‐miR‐200c‐3p intervention was conducive to HIF‐1α expression (Figure 6E‐G). The results of in vivo experiments implied that a circ_0007331/miR‐200c‐3p/HIF‐1α axis‐based regulatory pathway might exist in the endometrium of endometriosis, which could aggravate the endometriosis progression.

## 4 DISCUSSION

Although endometriosis has been identified as a debilitating disease with benign characteristics, the various symptoms that accompany it seriously disturb the quality of life of thousands of women of reproductive age. Due to the complexity of endometriosis symptoms associated with various diseases, there is no clear consensus on the pathological mechanism of this disease at the moment. Endometriosis is firstly defined as an inflammatory disease, characterized by the activation of macrophage migration inhibitory factor (MIF), tumour necrosis factor‐alpha (TNF‐α) and multiple interleukins (IL‐6, IL‐8, IIL‐1β) and the up‐regulation of inflammatory biomarkers including monocyte chemotactic protein 1 (MCP‐1) and C‐reactive protein (CRP).^18^ Additionally, endometriosis also exhibits cancer‐like characterizations such as neovascularization, cell invasion and unrestrained cell proliferation.^19^ The disease is not regarded as a malignant disease, while researchers have recognized the possibility of endometriotic tissues transforming into malignant ovarian cancer. Accurate detection and early diagnosis are crucial for the therapy of endometriosis, but owing to the diversity and concealment of its inchoate symptoms, the current gold standard for diagnosis still consists of invasive surgery and the following histopathology examination, which often results in 5‐10 years delay in the management of this disease. Therefore, the research in this field mainly focuses on the development of novel non‐invasive biomarkers from the biological fluids of patients for the early detection of diseases recently. The reported potential biomarkers for endometriosis usually originate from blood and urine, including miRNAs, lncRNAs, growth factors, glycoproteins and proteins related to immunity or angiogenesis. The representative glycoprotein served as a biomarker for endometriosis is CA‐125, which usually characterizes the degree of severity in this disease.^20^ The single levels of inflammatory cytokines IL‐1, IL‐6 and IL‐8 do not reflect the pathological development of endometriosis, but combined usage has the potential to mark diseases.^21^ The expression level of insulin‐like growth factor‐1 (IGF‐1) has been reported to be significantly different in stages I‐II‐IV and I‐II of endometriosis, but subsequent studies have added much controversy for its use as a biomarker.^22^ TC0101441 was found to exhibit a statistically significant positive correlation with infertility and chronic pelvic pain, which has the potential to diagnose the severity of the disease.^23^ Although the above‐mentioned biomolecules can more or less reflect the single‐dimensional physiological characteristics of endometriosis, due to the lack of specificity and sensitivity for this pathology, there is still a certain gap from clinical application. In recent years, miRNA has been predicted as an ideal biomarker for the diagnosis of endometriosis due to its advantages of high humoral stability and high tissue specificity. However, it has been found that no single miRNA could provide sufficient specificity and sensitivity to this disease after several further studies.

Mounting evidence showed that circRNAs can be adopted to assess the diagnosis and prognosis of various diseases and even as a therapeutic target, such as cancer, CNS disease, cardiovascular disease and certain endocrine dysfunction.^24^ However, there is little research on the biological function and molecular mechanism of circRNAs in endometriosis. Circ_0004712 and circ_0002198 are the earliest published novel biomarkers promising for the diagnosis of endometriosis.^12^ CircRNA_103237 was proved to suppress cell proliferation and invasion in inchoate secretory endometrium of women with endometriosis through sponge miR‐34.^25^ EMT, as a prerequisite for endometriosis, was found to be regulated by the signalling pathway hsa_circ_0067301/miR‐141/Notch.^17^ In this study, we identified aberrant high expression of circ_0007331 in endometriosis by comparing endometrial tissue samples from patients with endometriosis and normal individuals. Further in vitro functional experiments demonstrated that circ_0007331 can facilitate the viability, proliferation and invasion of endometrial cells in endometriosis. We then investigated the molecular mechanism of circ_0007331 in endometriosis and found that it may play the regulatory role in endometrial cells by sponging miR‐200c‐3p to regulate the HIF‐1α expression. Such information may promote new therapeutics for endometriosis in the future.

MiR‐200c‐3p originates from the miR‐200 family characterized as tumour suppressors, which consists of five highly conservative members including miR‐141, miR‐200a/200b/200c and miR‐429.^26^ In most cases, the miR‐200 family inhibits EMT, cell invasion and angiogenesis of tumours through enhancing the expression of E‐cadherin.^27^ Past research has confirmed that miR‐200c is essential in the life activity of the cells such as EMT, cancer cell metastasis and invasion, epigenetic regulation, stem cell pluripotency, cell growth and proliferation, chemosensitivity and programmed cell death and is related to cell transformation, tumorigenesis, tissue and organ fibrosis and other body dysfunction.^28^ In this study, we found that the expression level of miR‐200c‐3p is inversely proportional to the viability, proliferation and invasion ability of endometrial cells, which is consistent with the carcinoid characteristics of endometriosis and the anti‐cancer effect of miR‐200c.

HIF‐1α is a transcription factor present in human and mammalian cells under hypoxic conditions, which plays a vital role in mediating angiogenesis and promoting cell proliferation.^29^ Overexpression of HIF‐1α has been found in a variety of cancers, including pancreatic cancer, breast cancer, prostate cancer, colon cancer and lung cancer.^30^ Previous studies showed that the occurrence and development of endometriosis were closely related to local angiogenesis and hypoxic mechanisms. Further quantitative analysis proved that HIF‐1α protein levels in ectopic endometriosis tissues were higher than eutopic endometrial tissues.^31^ Growing evidence shows that HIF‐1α plays a key role in the development of ectopic endometrium by mediating genes related to oestrogen production, angiogenesis, proliferation and inflammation.^32^ Moreover, HIF‐1α inhibitors were gradually adopted in the therapeutic strategy for endometriosis like rapamycin, 2‐methoxyestradiol and HDAC inhibitors. However, previous research mainly focused on the functional study of HIF‐1α in endometriosis. Even if the mechanism was involved, the key point was often on the downstream signals rather than the upstream regulatory factors of HIF‐1α such as circRNAs or miRNAs. We reported the first circRNAs that affects the regulation of HIF‐1α on endometriosis and explored its possible mechanism. This not only provides a potential tool for other researchers to continue to explore the deeper mechanism of HIF‐1α in endometriosis, but also offers a choice for the therapeutic strategy of endometriosis targeting HIF‐1α, which is different from small molecule inhibitors.

However, our research remains a key question. The initial aim of this work was to search for a non‐invasive biomarker for the diagnosis of endometriosis. Unfortunately, we only evaluated and investigated the differential distribution and biological function of circ_0007331 in ectopic tissues. Although circ_0007331 has considerable potential as a therapeutic target, it needs further studies in peripheral blood or menstrual blood for the clinical applications as a non‐invasive diagnostic biomarker.

In summary, our results show that circ_0007331 is abnormally highly expressed in patients with endometriosis. Both in vitro or in vivo, circ_0007331 knock‐down suppresses the progression of endometriosis by inhibiting the proliferation and invasion of ectopic endometrial cells. Further mechanism studies confirmed that the molecular basis of this function is the circ_0007331/miR‐200c‐3p/HIF‐1α axis. Our work not only reveals the potential therapeutic targets for future drug interventions in endometriosis, but also provides novel insights for the fine regulation of HIF‐1α in endometriosis.

## CONFLICT OF INTEREST

No potential conflict of interest was reported by the authors.

## AUTHOR CONTRIBUTION

**Lan Dong:** Data curation (equal); Investigation (equal); Supervision (equal); Validation (equal); Visualization (equal); Writing‐original draft (lead). **Lu Zhang:** Data curation (equal); Investigation (equal); Validation (equal). **Hua Liu:** Investigation (equal); Software (equal); Visualization (equal); Writing‐original draft (equal). **Meiting Xie:** Software (equal). **Jing Gao:** Validation (equal). **Xiaoyan Zhou:** Visualization (equal). **Qinghong Zhao:** Conceptualization (equal); Investigation (equal); Methodology (equal). **Silin Zhang:** Conceptualization (equal); Investigation (equal); Resources (equal). **Jing Yang:** Conceptualization (equal); Investigation (equal); Project administration (lead); Resources (equal); Writing‐review & editing (lead).

## Data Availability

The data used to support the findings of this study are available from the corresponding author upon reasonable request.
